# 

**Published:** 1883-06

**Authors:** 


CONTENTS:

ART. I.—The Application of Disinfectants and Escharotics in the Treatment of Alveolar Abscess. By Dr. Frank Brewer........ 49

ART. II.—Continuous Gum Work. By A B. Verrier, L. D. S ........ 56

ART. III—Should Anæsthesia be Resorted to for the Extraction of Teeth. By J. H. Coyle, D. D. S........ 64

ART. IV.—Periodontitis—Cause and Treatment. B Dr. J. Campbell........ 71

ART. V.—Dental Caries. By Dr. W. D. Miller........ 77

ART VI.—Amalgam Fillings ........ 83

ART. VII —Metallic Crowns. By Wm. N. Morrison, D. D. S........ 86

Southern Dental Association........ 88

Pennsylvania State Dental Society........ 89

EDITORIAL, ETC.

The Composition and Effects of Chloral Hydrate........ 89

MONTHLY SUMMARY.

The Care of the Teeth........ 91

Under the Surgeon's Touch........ 93

A Rare Case of Exostosis........ 94

Foreign Bodies in the Air Passages........ 94

Hardening Plaster Casts ........ 95

Our Dental Brethern........ 95

A Simple Method of Producing Local Anæsthesia........ 96

Dental Formation in the Nasal Cavity........ 96

